# Meteorological Table

**Published:** 1811-06

**Authors:** 


### METEOROLOGICAL TABLE.

*From April 27, to May 27.*

| | D | Therm. | | | Barom. | | | Hygrom. dry——damp | | | Atmospherical Variations |
|---|---|---|---|---|---|---|---|---|---|---|---|
| | 27 | 54 | 61 | 54 | 29$^{5}$ | — | —$^{6}$ | 28 | 10 | — | E .. F .... |
| | 28 | 51 | 62 | 55 | 29$^{6}$ | — | —$^{5}$ | 21 | 5 | 15 | S . F .. — .... R .. R . in n. |
| | 29 | 53 | 52 | 49 | —$^{4}$ | — | —$^{6}$ | 27 | 33 | — | W ... F .. R ... Hail .. C .. |
| ◐ | 30 | 51 | — | 50 | —$^{7}$ | — | — | 40 | — | — | W ... R . R .. in night |
| | 1 | 55 | 60 | 57 | —$^{6}$ | — | — | 57 | 80 | 77 | S .. R .. SW ... R .. |
| | 2 | 56 | 59 | 53 | —$^{6}$ | — | —$^{9}$ | 75 | 67 | 56 | SW .. C .... R .. C .... |
| | 3 | 53 | 57 | 55 | 30 | — | 29$^{8}$ | 62 | 52 | 70 | SW .. C .... R . — ... in n. |
| | 4 | 56 | 62 | 57 | —$^{8}$ | 30 | 29$^{9}$ | 79 | 72 | — | W ... C .... F .. R ... in n. |
| | 5 | 56 | 59 | 53 | —$^{7}$ | —$^{6}$ | —$^{9}$ | 80 | 63 | 53 | SW . R .. C .. R . |
| | 6 | 49 | 53 | 49 | 30 | — | 29$^{9}$ | 50 | 59 | — | E .. C ... R .. — ... in n. |
| | 7 | 53 | — | 52 | —$^{7}$ | — | —$^{9}$ | 68 | 66 | 68 | W .. C . R ... F . |
| ○ | 8 | 50 | 57 | 53 | —$^{7}$ | — | —$^{6}$ | 67 | 50 | 70 | E ..C .... SE .. F . R ... in n. |
| | 9 | 54 | 53 | 52 | —$^{5}$ | — | —$^{7}$ | 71 | 75 | 70 | SE .. R .. — ... F .... R ... |
| | 10 | 52 | 61 | 56 | —$^{7}$ | —$^{8}$ | —$^{7}$ | 71 | 55 | 65 | SW .. F . — ... R .. |
| | 11 | 56 | 64 | 62 | —$^{8}$ | — | — | 66 | 42 | 56 | SW .. R . F ... — .... |
| | 12 | 62 | 75 | 69 | —$^{7}$ | —$^{5}$ | —$^{6}$ | 60 | 20 | 30 | S .. F .... |
| | 13 | 66 | 74 | 69 | —$^{5}$ | — | — | 39 | 20 | 14 | S .. F .. — .... |
| | 14 | 68 | 65 | 66 | —$^{5}$ | —$^{6}$ | —$^{7}$ | 15 | 10 | 17 | SW .. F .. — .... |
| ◑ | 15 | 58 | 68 | 61 | —$^{8}$ | — | —$^{7}$ | 22 | 3 | 17 | E ... F .... — R .. |
| | 16 | 62 | 67 | 59 | —$^{8}$ | — | — | 26 | 14 | 11 | NE .. C ... F ... C ... |
| | 17 | 58 | 65 | — | —$^{8}$ | — | —$^{9}$ | 34 | 7 | 6 | NE .. F ... — |
| | 18 | 66 | 67 | 66 | —$^{8}$ | — | — | 16 | 20 | 22 | SE .. F ... — C .. |
| | 19 | 63 | — | — | —$^{9}$ | — | — | 45 | — | — | N .. R ... — C .... |
| | 20 | 58 | 73 | 60 | —$^{9}$ | —$^{8}$ | —$^{7}$ | 43 | 24 | 30 | NE .. F .. — .... C .. |
| | 21 | 56 | — | 61 | —$^{7}$ | — | — | 42 | 57 | — | NE .. R .... C .... |
| ● | 22 | 61 | 70 | 64 | —$^{7}$ | —$^{6}$ | — | 60 | 52 | 57 | NE .. C ... F ... R ... |
| | 23 | 57 | 65 | — | —$^{8}$ | —$^{9}$ | — | 49 | 54 | — | W ... F .. NW ... F ... |
| | 24 | 60 | 63 | — | —$^{9}$ | —$^{8}$ | — | 23 | 28 | — | S . R ... F .. |
| | 25 | 64 | 71 | 63 | 30 | — | — | 36 | 11 | 19 | S .. F ... — .... — ... |
| | 26 | 64 | 75 | 70 | 30 | — | — | 27 | 16 | 20 | W . F . — .... C .. |
| | 27 | 65 | 75 | 62 | 30 | 29$^{9}$ | —$^{7}$ | 30 | 22 | — | W .. C .. F .... R .. E . |

11th. Lightning in the evening from the North.

20th. Lightning in the South, with distant thunder at nine in the evening. Thunder storm in the night with heavy rain.

22d. Thunder storm from the South, passing over the metropolis, and falling heavy on the city near St. Paul's. Reports of damage done by this storm. A gentleman ascending Highgate Hill was struck from his horse by the lightning. Distant thunder had been heard during the day.

26th. Lightning in the evening in the East, with distant thunder.

Quantity of Rain from April 27 to May 27, 2 inches and $\frac{43}{100}$.

The hygrometer during the whole of this month has indicated an unusual degree of humidity in the atmosphere; and a great quantity of rain has fallen. The warm and humid state of the air has occasioned vegetation to be extremely luxuriant.

*Princes Street, Cavendish Square*

